# Antioxidant and Antimicrobial Properties of Selected Fruit Juices

**DOI:** 10.1007/s11130-022-00983-2

**Published:** 2022-07-13

**Authors:** Dariusz Nowak, Michał Gośliński, Lucyna Kłębukowska

**Affiliations:** 1 Department of Nutrition and Dietetics, Faculty of Health Sciences, Ludwik Rydygier Collegium Medicum in Bydgoszcz, Nicolaus Copernicus University in Toruń, Dębowa 3, 85-626 Bydgoszcz, Poland; 2 Department of Industrial and Food Microbiology, Faculty of Food Science, University of Warmia and Mazury in Olsztyn, pl. Cieszyński 1, 10-726 Olsztyn, Poland

**Keywords:** Berry juices, Vitamin C-rich juices, Antioxidant properties, Antimicrobial activity

## Abstract

Fruit and fruit juices are a valuable source of bioactive compounds, which can protect our organisms from oxidative stress. The phenolic compounds and other phytochemicals may affect the antimicrobial properties of juices. The aim of this study has been to evaluate antioxidant and antimicrobial properties of selected berry juices and vitamin C-rich fruit juices. The research material was composed of seven juices, including three from berries (elderberry chokeberry, cranberry), three from vitamin C-rich fruit (sea buckthorn, wild rose, Japanese quince) and one exotic juice from noni fruit. Antioxidant capacity, total polyphenol, total flavonoid and total anthocyanin content were determined. Furthermore, the antimicrobial activity and the minimal inhibitory concentration (MIC) as well as the minimal bactericidal concentration (MBC) were evaluated. The research showed that fruit juices from wild rose, chokeberry and Japanese quince had the highest antioxidant capacity. These juices were characterised by the rich content of polyphenols. Elderberry and chokeberry juices had the highest total anthocyanins. The juices differed in the content of bioactive compounds and specific bactericidal properties against Gram-positive or Gram-negative bacteria. Fruit juices from cranberry, Japanese quince and sea buckthorn had the highest antimicrobial activity. Wild rose, chokeberry and elderberry juices, despite their high antioxidant properties, showed antimicrobial activity only against Gram-positive strains, except *Enterococcus faecalis* and *Clostridium perfringens*. Significant differences in the content of bioactive compounds in fruit juices affect the antimicrobial properties juices.

**Supplementary Information:**

The online version contains supplementary material available at 10.1007/s11130-022-00983-2.

## Introduction

Consumption of fruits and vegetables is essential in diversified and nutritious diet, which according to WHO recommendations should contain at least 400 g *per *day (5 servings). One portion may be replaced by a glass of good quality juice. Furthermore, fruit and fruit juices are consumed for their taste and aroma, but they are also rich in bioactive compounds, which can protect us from oxidative stress. Other than citrus fruit, popular all over the world [1], another source of valuable bioactive substances are berry fruits, mainly chokeberry, elderberry, cranberry, etc., which are abundant in phenolic compounds, mostly flavonoids and phenolic acids [2–4]. Other sources of phytochemicals are searched for among exotic fruit or vitamin C-rich fruit and fruit juices [5, 6]. The studies showed that vitamin C-rich juices can contain from about 2000 mg (Japanese quince) to over 3000–4000 mg of ascorbic acid *per* kilogram (sea buckthorn and wild rose) [6, 7]. Consumption of fruit juices is beneficial for the maintenance of good health and prevention of non-communicable chronic diseases, such as cardiovascular diseases, cancers, and neurodegenerative disease. Bioactive compounds such as polyphenols and vitamins have antioxidative, anti-inflammatory, antitumor, and antimicrobial activities [8–10]. It has been reported that concentrates of citrus juices may have a beneficial antimicrobial effect related to the presence of various compounds with antioxidant properties [10]. Berry juices and extracts may have also antimicrobial activity [11]. It has been shown that berry polyphenols are capable of selectively inhibiting the growth of different human pathogenic bacteria [12, 13]. The antibacterial activity of juices derived from blackcurrant, redcurrant, cranberry and raspberry on common oral pathogenic Gram-positive and Gram-negative bacteria has also been investigated, leading to varied results [14]. Some exotic fruit may present similar properties. For example, noni fruit (*Morinda citrifolia* L.) contains compounds that can act as antibacterial agents [15]. Fruit phytochemicals can directly inhibit bacterial growth or act indirectly by modulating the expression of virulence factors, both of which reduce microbial pathogenicity [16]. Thus, the search for new antimicrobial compounds from natural sources continues [10, 17], which is very important considering the resistance of numerous pathogens to pharmaceuticals. Both juices from regional berries (chokeberry, elderberry or cranberry) and vitamin C-rich juices (wild rose, Japanese quince or sea buckthorn) or of exotic origin (noni berries) can be sources of natural bioactive compounds. However, there is a lack of comprehensive studies assessing the effect of juices and their bioactive compounds on antimicrobial properties against Gram-positive and Gram-negative bacteria. Considering the above, the aim of this study has been to evaluate the antioxidant and antimicrobial activities of selected berry juices and vitamin C-rich fruit juices.

## Materials and Methods

The material and methods section is presented as supplementary material 1.

## Results and Discussion

All the analysed fruit juices were sour and had pH between 2.5 and 3.8 (Table 1). The lowest pH values were determined in cranberry juice (2.5), followed by Japanese quince and sea buckthorn juices (2.8). The highest pH value noted in our study (3.8) was determined in elderberry, wild rose and noni juices. In other studies, the values of pH were similar, ranging from 2.4 to 2.9 for Japanese quince juice [18], 2.91 to 3.0 for sea buckthorn fruits [19] and about 2.5 for cranberry juice [6]. The high acidity of cranberry juice is due to the high content of vitamin C, at 897 mg L^−1^ [20].

**Table 1:** Antioxidant properties of the analysed fruit juices

	Elderberry	Chokeberry	Cranberry	Wild rose	Japanese quince	Sea buckthorn	Noni
pH	3.8 ± 0.2^a^	3.6 ± 0.1^a^	2.5 ± 0.1^c^	3.8 ± 0.2^a^	2.8 ± 0.1^b^	2.8 ± 0.1^b^	3.8 ± 0.1^a^
Soluble solids content (%)	10.0 ± 0.5^b^	15.0 ± 0.5^a^	8.5 ± 0.5^c^	15.5 ± 1.0^a^	7.5 ± 0.5^c^	8.0 ± 0.5^c^	7.0 ± 0.5^c^
DPPH [mg Tx L^−1^]	2159 ± 17^d^	4853 ± 159^b^	904 ± 2^e^	5532 ± 194^a^	3762 ± 38^c^	963 ± 4^e^	907 ± 2^e^
ABTS [mg Tx L^−1^]	3053 ± 143^b^	6978 ± 246^a^	1030 ± 17^c^	6972 ± 349^a^	6451 ± 145^a^	745 ± 24^d^	1375 ± 49^c^
TP [mg GAE L^−1^]	6826 ± 171^c^	11,266 ± 475^b^	2773 ± 49^e^	16,619 ± 401^a^	11,018 ± 421^b^	4701 ± 46^d^	3005 ± 108^e^
FBBB [mg GAE L^−1^]	7857 ± 46^b^	11,483 ± 20^a^	2239 ± 110^d^	5967 ± 42^c^	6375 ± 45^c^	11,524 ± 59^a^	1270 ± 82^e^
TF [mg CAE L^−1]^	804 ± 14^c^	5069 ± 14^a^	24 ± 1^d^	5607 ± 117^a^	3214 ± 118^b^	3833 ± 88^b^	< 1^e^
TA (mg CGE L^−1^)	1966.8 ± 18^a^	1209.7 ± 3^b^	712.5 ± 1^c^	0.3 ± 0.0^d^	0.3 ± 0.0^d^	534.4 ± 2^c^	0.1 ± 0.0^d^

Data are mean ± standard deviation (*n* = 3). Statistical analysis was performed by one-way ANOVA using the Tukey’s *post hoc* test: different letters in the same row indicate statistical significance (*p* ≤ 0.05)

*Abbreviations*: *DPPH* 1,1-diphenyl-2-picrylhydrazyl, *ABTS* 2,2′-azino-di-[3ethylbenzthiazoline sulphonate], *TP* total polyphe-nol content, *FBBB* 4-benzoylamino-2,5-diethoxybenzenediazo-nium chloride hemi(zinc chloride) salt, *TF* total flavonoid content, *TA* total anthocyanins, *Tx* trolox equivalents, *GAE* gallic acid equivalents, *CAE* catechin equivalents, *CGE* cyanidin-3-mono-glucoside equivalents

Next, the antioxidant properties of all the tested fruit juices were determined (Table 1). The antioxidant capacity (DPPH assay) was the highest for wild rose juice (5532 ± 194 mg Tx L^−1^). A relatively high value was determined for chokeberry juice (4853 ± 159 mg Tx L^−1^). Slightly lower antioxidant properties were observed in Japanese quince juice (3762 ± 38 mg Tx L^−1^). The lowest antioxidant capacity was demonstrated by cranberry juice, noni juice and sea buckthorn juice (from 904 ± 2 to 963 ± 4 mg Tx L^−1^). The antioxidant capacity measured with the ABTS method confirmed the parallel results obtained with the DPPH method. In this approach, higher antioxidant capacity was determined in juices from wild rose, chokeberry and Japanese quince (from 6451 ± 145 to 6978 ± 246 mg Tx L^−1^). However, in contrast to the DPPH results, the ABTS method showed that the antioxidant capacity of these three juices did not differ statistically significantly at *p* ≤ 0.05. Juices with the highest antioxidant capacities (determined in DPPH and ABTS assay) had the highest content of total polyphenols determined by the Folin-Ciocalteu method (Table 1). Wild rose juice had statistically significant the highest total polyphenol content (16,619 ± 401 mg GAE L^−1^). Chokeberry and Japanese quince juices also contained a large amount of polyphenols, however they did not differ statistically significantly (TP 11,266 ± 475 and 11,018 ± 421 mg GAE L^−1^, respectively). The other juices were also a good source of polyphenols, but their content of these compounds was much lower, except elderberry juice (6826 ± 171 mg GAE L^−1^). The FBBB method yielded slightly different results. The highest total polyphenol content was determined in sea buckthorn juice (11,524 ± 59 mg GAE L^−1^), followed by chokeberry juice (11,483 ± 20 mg GAE L^−1^). Elderberry juice had a much lower amount of polyphenols, although slightly higher than determined in Japanese quince and wild rose juices. The lowest total polyphenol content, same as found with the Folin-Ciocalteu assay, was determined in noni and cranberry juices (1270 ± 82 and 2239 ± 110 mg GAE L^−1^, respectively). Wild rose and chokeberry juices also contained the highest total flavonoid content (5607 ± 117 and 5069 ± 14 mg CAE L^−1^, respectively). Much lower values were determined in juices from sea buckthorn and from Japanese quince (3833 ± 88 and 3214 ± 118 mg CAE L^−1^). Noni and cranberry juices had the smallest amount of flavonoids, which correlated with their lowest content of polyphenols. Elderberry juice was found to be the richest source of anthocyanins (1966.8 ± 18 mg CGE L^−1^), followed by chokeberry juice (1209.7 ± 3 mg CGE L^−1^). Much lower amounts of anthocyanins were determined in cranberry and sea buckthorn juices (712.5 ± 1 and 534.4 ± 2 mg CGE L^−1^ respectively). The other juices contained trace levels of anthocyanins.

Furthermore, we determined the correlation between the analytical methods used. The correlation coefficients (R^2^) for antioxidant properties of analysed juices ranged from 0.095 to 0.976 (Table 2). DPPH and ABTS assays showed a high correlation coefficient (R^2^ = 0.976). In addition, both methods demonstrated a high linear correlation with TP (0.972 and 0.929, respectively), which confirms that polyphenols contributed more to antioxidant capacity than other compounds, for example anthocyanins. The lowest correlation coefficients were determined for TA (from 0.088 to 0.095), which demonstrated that anthocyanins were minor contributors of the antioxidant properties of analysed juices. On the other hand, a low correlation was found between the antioxidant capacity (DPPH and ABTS assay) and FBBB.

**Table 2:** The correlation coefficients (R^2^)

	DPPH	ABTS	TP	FBBB	TF	TA
DPPH		0.976	0.972	0.347	0.803	0.088
ABTS	0.976		0.929	0.312	0.728	0.095
TP	0.972	0.929		0.334	0.826	0.173
FBBB	0.347	0.312	0.334		0.679	0.421
TF	0.803	0.728	0.826	0.679		0.184
TA	0.088	0.095	0.173	0.421	0.184	

*Abbreviations*: *DPPH* 1,1-diphenyl-2-picrylhydrazyl, *ABTS* 2,2′-azino-di-[3ethylbenzthiazoline sulphonate], *TP* total polyphe-nol content, *FBBB* 4-benzoylamino-2,5-diethoxybenzenediazo-nium chloride hemi(zinc chloride) salt, *TF* total flavonoid content, *TA* total anthocyanins, *Tx* trolox equivalents, *GAE* gallic acid equivalents, *CAE* catechin equivalents, *CGE* cyanidin-3-mono-glucoside equivalents

In this study, wild rose, chokeberry and Japanese quince were characterised by the highest antioxidant properties. These juices had the highest antioxidant capacity (both DPPH and ABTS assays) as well as the highest total polyphenol content and the highest total flavonoids. Nadpal et al. [21] also reported high antioxidant properties (DPPH, total phenolics and total flavonoid) of wild rose (*Rosa canina*). The high content of polyphenols, especially flavonoids, in different wild rose varieties has been demonstrated by Roman et al. [22] and Aleksashina et al. [23]. Another study has shown that plant extracts from *R. canina,* containing numerous flavonoids, led to a considerable decrease of reactive oxygen species (ROS) in the human endothelium. The authors concluded that extracts of *R. canina* could be a valuable ingredient of many supplements intended to alleviate oxidative stress [24]. Our study showed that also chokeberry juice had high antioxidant properties. It contained significant amounts of phenolic compounds, mostly flavonoids, as well as anthocyanins. The total polyphenol content in chokeberry juice was similar to that reported by Tolić et al. [25], although the content of flavonoids and anthocyanins was lower. The authors pointed that the differences in the content of polyphenols were related to plant growing conditions (*e.g*., temperature, exposure to the sun, rainfalls). Furthermore, differences in the composition of bioactive compounds may arise from different juice production technologies. The chokeberry anthocyanin profile is very simple, consisting mainly of cyanidin glycosides, such as cyanidin-3-*O*-arabinoside, cyanidin-3-*O*-galactoside, cyanidin-3-*O*-glucoside, and cyanidin-3-*O*-xyloside [26]. In our study, chokeberry juice had less of anthocyanins than elderberry juice. Kaack and Austed [26] reported that the major elderberry anthocyanins were cyanidin-3-glucoside and cyanidin-3-sambubioside. Besides wild rose and chokeberry juices, Japanese quince juice showed high antioxidant properties. This juice was characterised by a high quantity of total polyphenol but only a trace amount of anthocyanins. Other authors likewise reported high antioxidant properties of Japanese quince [18, 27]. It is claimed that 94% of the total polyphenols in Japanese quince is composed of flavan-3-ol, mainly (−)-epicatechin, followed by (+)-catechin. This fruit also contains smaller quantities of chlorogenic acid, rutin and isoquercitin [7]. Another important compound found in Japanese quince is vitamin C amount in this fruit can be as high as 1700–2000 mg AA kg^−1^ [6, 7]. The other juices analysed in our study had lower antioxidant properties. However, the high content of polyphenols and flavonoids in sea buckthorn juice was noteworthy. Criste et al. [28] reported that sea buckthorn fruit are a rich source of phenolic compounds, especially quercetin derivatives and hydrocinnamic acid derivatives. The content of total polyphenols and flavonoids was 2- to 3-fold higher in leaf than in berry extracts. Moreover, the antioxidant properties of sea buckthorn arise from its content of other phytochemicals, *e.g*., carotenoids, such as lutein, zeaxanthin, *β*-cryptoxanthin, *cis*-*β*-carotene, and *β*-carotene [28]. Sea buckthorn juice also contained some amount of anthocyanins, same as cranberry juice. It has been shown that cranberries are rich in several groups of flavonoids, particularly proanthocyanidins, anthocyanidins, and flavonols, together with phenolic acids and benzoates [29, 30]. Furthermore, we have noted that the results of antioxidant capacity of juices were higher in the ABTS assays than in the DPPH ones. It has been shown that ABTS^+^ radicals are more reactive than DPPH radicals, and reactions with ABTS^+^ radicals involve a single-electron transfer process and flavonoids, like quercetin derivatives, are able to chelate free radicals immediately by single-electron transfer [28, 31]. The antioxidant capacity (DPPH and ABTS assay) analysed juices was high correlated with the total polyphenol content but low correlated with FBBB assay and total anthocyanins. Whereas in the study Narwojsz et al. [32], the antioxidant capacity was correlated with the content of phenolic compounds but also with flavonoids and proanthocyanidins.

### Antimicrobial Activity of Juices

The analysed juices showed varied antimicrobial activity against the tested bacterial strains, manifested by the diameters of the growth inhibition zones (Table 3). The results distinctly show that higher antimicrobial activity was achieved against Gram-positive strains, where the juices inhibited the growth of 50 to 100% of the strains. The most resistant strains among these bacteria were *Enterococcus* and *Clostridium perfringens*. The highest antimicrobial activity against the tested Gram-positive bacteria was demonstrated by sea buckthorn, Japanese quince and cranberry juices. These were also the only juices that showed antimicrobial activity against all tested strains of Gram-negative bacteria. Our results coincide with the data provided by Haghayeghi et al. [33], who achieved higher antimicrobial effects against Gram-positive strains than against Gram-negative strains. Kranz et al. [14] analysed the antimicrobial activity of various berry juices. They concluded that cranberry juice, like black and red current juices, demonstrated antimicrobial activity against the tested strains. In contrast, our study showed that elderberry and chokeberry juices presented no such activity against Gram-negative strains. Another study [34] reported that cranberry, noni, goji and sea buckthorn juices had the inhibitory effect of the growth of the tested strains. The authors showed that cranberry juice had a high antimicrobial activity against Gram-positive strains, which was similar to our results. However, noni juice had a strong antimicrobial effect on Gram-positive bacterial strains, which was not confirmed in our study. The cited authors suggested that the major antimicrobial factor was the acidic pH of tested fruit juices [34]. Urbanaviciute et al. [7] showed the antimicrobial activity of all Japanese quince extracts against Gram-positive and Gram-negative strains, which was confirmed in our research. Similarly, sea buckthorn extract was characterised by a high antimicrobial effect on all the analysed Gram-positive and Gram-negative strains [28]. All extracts showed antibacterial activity against *Staphylococcus aureus*, *Bacillus cereus*, and *Pseudomonas aeruginosa*, although extracts from berries were less potent than extracts from leaves [28].

**Table 3:** Antimicrobial activity of juices against bacterial strains tested by diffusion assay

Tested juice	Zone of inhibition [mm]											
	Gram-positive test strains											
	*Staphylococcus aureus*			*Enterococcus faecalis*			*Listeria monocytogenes*		*Listeria inocua*	*Bacillus cereus*		*Clostridium perfringens*
	*G3*	*2G*	*01*	*24*	*11*	*07*	*67*	*74*	*LI0001*	*1*	*9*	*Clpe0001*
Elderberry	0	0	18 ± 0.9^d^	0	0	0	21 ± 0.5^d^	22 ± 1.2^d^	17 ± 0.5^d^	18 ± 4.3^de^	17 ± 0.0^c^	0
Chokeberry	22 ± 0.5^c^	21 ± 0.5^c^	20 ± 0.9^d^	0	0	0	24 ± 1.2^c^	25 ± 0.5^c^	23 ± 1.6^c^	23 ± 0.5^c^	25 ± 0.5^b^	0
Cranberry	31 ± 3.3^a^	30 ± 0.0^b^	31 ± 0.5^b^	30 ± 0.8^a^	24 ± 1.2^b^	30 ± 0.8^a^	31 ± 1.4^b^	32 ± 0.9^b^	34 ± 0.9^a^	26 ± 0.9^b^	30 ± 1.2^a^	24 ± 1.2^a^
Wild rose	32 ± 1.4^a^	21 ± 0.0^c^	26 ± 3.7^c^	0	0	0	24 ± 0.5^c^	23 ± 2.2^d^	25 ± 0.8^b^	20 ± 0.5^d^	0	0
Japanese quince	35 ± 3.3^a^	38 ± 0.5^a^	38 ± 1.0^a^	26 ± 0.9^b^	27 ± 0.0^a^	26 ± 1.0^b^	37 ± 1.2^a^	36 ± 0.5^a^	35 ± 0.0^a^	32 ± 1.4^a^	30 ± 0.0^a^	21 ± 0.9^b^
Sea buckthorn	28 ± 1.2^b^	28 ± 1.2^b^	22 ± 0.0^d^	24 ± 1.2^b^	24 ± 0.5^b^	23 ± 0.5^c^	30 ± 0.0^b^	27 ± 2.5^c^	27 ± 0.0^b^	23 ± 0.0^c^	27 ± 0.5^b^	20 ± 0.5^b^
Noni	21 ± 0.9^c^	22 ± 2.8^c^	21 ± 0.9^d^	0	0	0	20 ± 2.1^d^	21 ± 0.9^d^	23 ± 1.2^c^	17 ± 0.0^e^	17 ± 0.5^c^	0
	Gram-negative test strains											
	*Escherichia coli*				*Klebsiella pneumonie*		*Salmonella typhimurium*		*Salmonella enteritidis*	*Pseudomonas aeruginosa*		*Pseudomonas fluorescens*
	*31*	*22*	*26*	*34*	*003*		*63 s*	*235*	*61 s*	*PA0001*		*ATCC13625*
Elderberry	0	0	0	0	0		0	0	0	0		0
Chokeberry	0	0	0	0	0		0	0	0	0		0
Cranberry	25 ± 2.9^a^	26 ± 0.9^a^	25 ± 0.0^a^	26 ± 0.9^a^	23 ± 0.5^a^		27 ± 0.5^a^	26 ± 0.5^a^	25 ± 0.9^a^	24 ± 0.9^a^		24 ± 0.9^b^
Wild rose	0	0	0	20 ± 0.0^c^	0		27 ± 0.0^a^	25 ± 2.4^a^	0	0		0
Japanese quince	26 ± 2.9^a^	25 ± 0.0^a^	27 ± 1.6^a^	26 ± 1.2^a^	23 ± 2.5^a^		27 ± 0.9^a^	25 ± 0.5^a^	22 ± 2.1^b^	25 ± 0.0^a^		24 ± 0.9^b^
Sea buckthorn	26 ± 0.5^a^	22 ± 0.5^b^	24 ± 1.6^a^	24 ± 0.0^b^	21 ± 0.9^a^		24 ± 0.5^b^	25 ± 0.5^a^	22 ± 1.7^b^	23 ± 0.9^a^		27 ± 2.1^a^
Noni	0	0	0	0	0		0	0	0	0		0

Statistical analysis was performed by one-way ANOVA using the Tukey’s *post hoc* test: different letters in the same column indicate statistical significance (*p* ≤ 0.05)

In our study, the antimicrobial activity of juices was determined following their neutralisation. The results presented in Table 4 showed that Japanese quince and cranberry juices were characterised by the highest antimicrobial activity against the tested strains of bacteria. Both samples of neutralised juices inhibited the growth of all Gram-positive and most of Gram-negative bacterial strains. None of the analysed juices showed antimicrobial activity against strains of *Klebsiella pneumonie*, and only sea buckthorn juice was effective against strains of *Pseudomonas fluorescens*.

**Table 4:** Antimicrobial activity of neutralized juices against bacterial strains tested by diffusion assay

Tested juice	Zone of inhibition [mm]											
	Gram-positive test strains											
	*Staphylococcus aureus*			*Enterococcus faecalis*			*Listeria monocytogenes*		*Listeria inocua*	*Bacillus cereus*		*Clostridium perfringens*
	*G3*	*2G*	*01*	*24*	*11*	*07*	*67*	*74*	*LI0001*	*1*	*9*	*Clpe0001*
Elderberry	0	0	16 ± 1.6^c^	0	0	0	18 ± 1.2^c^	19 ± 0.5^c^	15 ± 0.9^c^	17 ± 0.9^c^	17 ± 1.2^c^	0
Chokeberry	19 ± 0.5^c^	19 ± 1.2^c^	17 ± 1.6^c^	0	0	0	20 ± 0.5^c^	19 ± 0.8^c^	20 ± 0.8^b^	21 ± 0.5^b^	20 ± 0.8^b^	0
Cranberry	20 ± 1.6^c^	21 ± 1.7^b^	20 ± 0.0^b^	20 ± 1.2^a^	20 ± 1.2^a^	23 ± 1.2^a^	20 ± 0.5^c^	20 ± 0.9^c^	22 ± 1.2^b^	20 ± 0.0^b^	20 ± 0.9^b^	20 ± 0.0^a^
Wild rose	22 ± 2.2^c^	12 ± 2.1^d^	14 ± 0.0^d^	0	0	0	13 ± 0.9^d^	13 ± 0.9^e^	0	0	0	0
Japanese quince	32 ± 0.9^a^	30 ± 0.0^a^	30 ± 0.5^a^	22 ± 1.2^a^	18 ± 0.0^b^	18 ± 0.0^b^	28 ± 0.9^a^	30 ± 0.5^a^	24 ± 0.0^a^	25 ± 0.9^a^	22 ± 0.0^b^	17 ± 0.5^b^
Sea buckthorn	25 ± 0.9^b^	23 ± 0.5^b^	20 ± 0.9^b^	0	0	0	25 ± 0.0^b^	25 ± 0.5^b^	25 ± 0.9^a^	22 ± 0.9^b^	25 ± 0.5^a^	0
Noni	0	0	0	0	0	0	19 ± 0.5^c^	17 ± 0.9^d^	20 ± 0.0^b^	0	0	0
	Gram-negative test strains											
	*Escherichia coli*				*Klebsiella pneumonie*		*Salmonella typhimurium*		*Salmonella enteritidis*	*Pseudomonas aeruginosa*		*Pseudomonas fluorescens*
	*31*	*22*	*26*	*34*	*003*		*63 s*	*235*	*61 s*	*PA0001*		*ATCC13625*
Elderberry	0	0	0	0	0		0	0	0	0		0
Chokeberry	0	0	0	0	0		0	0	0	0		0
Cranberry	24 ± 0.9^a^	22 ± 0.0^a^	22 ± 0.5^a^	19 ± 1.2^a^	0		20 ± 1.2^b^	22 ± 0.5^a^	20 ± 1.7^a^	0		0
Wild rose	0	0	0	0	0		0	14 ± 0.5^b^	0	0		0
Japanese quince	0	0	22 ± 0.5^a^	20 ± 0.9^a^	0		24 ± 1.7^a^	22 ± 0.0^a^	20 ± 0.0^a^	23 ± 0.9^a^		0
Sea buckthorn	0	0	0	20 ± 0.9^a^	0		20 ± 0.5^b^	19 ± 1.2^a^	22 ± 0.9^a^	21 ± 0.9^a^		20 ± 0.5^a^
Noni	0	0	0	0	0		0	0	0	0		0

Statistical analysis was performed by one-way ANOVA using the Tukey’s *post hoc* test: different letters in the same column indicate statistical significance (*p* ≤ 0.05)

The MIC (with the macrodilution method) and MBC were determined for the three juices whose antimicrobial activity determined with the diffusion method was the highest (Table 5). In this part of our study, we were also able to confirm the literature data that the determined values of the MBC were higher than the MIC values. The results were slightly varied depending on which juice was tested. In this stage cranberry juice was the most effective as it inhibited the growth of the tested strains at the lowest concentration. Our study confirmed the results obtained by Stobnicka et al. [34], who showed that cranberry juice most effectively inhibited the tested strains of bacteria. In another research, the antimicrobial activity of sea buckthorn extracts was assessed by determining the minimum inhibitory concentration (MIC). All extracts showed some level of antimicrobial activity against the target Gram-positive bacteria: *S. aureus*, *B. cereus*, and Gram-negative bacterium: *P. aeruginosa* [28].

**Table 5:** Results of antibacterial activity of juices (MIC, MBC) on tested bacteria strains

Tested strain	Cranberry juice		Japanese quince juice		Sea buckthorn juice	
	MIC	MBC	MIC	MBC	MIC	MBC
	[mg/mL]					
*Staphylococcus aureus* G3	2.0 ^a^	2.0 ^A^	4.0 ^b^	4.7 ^B^	4.0 ^b^	4.8 ^B^
*S. aureus* 2G	2.0 ^a^	2.0 ^A^	4.0 ^b^	4.8 ^B^	4.8 ^c^	4.8 ^B^
*S. aureus* 01	2.0 ^a^	2.8 ^A^	4.0 ^b^	4.8 ^B^	4.0 ^b^	4.8 ^B^
*Enterococcus faecalis* 24	4.0 ^a^	4.8 ^A^	7.8 ^b^	9.7 ^C^	7.8 ^b^	8.1 ^B^
*E. faecalis* 11	4.8 ^a^	8.1 ^A^	7.8 ^b^	13.7 ^B^	9.7 ^c^	13.7 ^B^
*E. faecalis* 07	4.8 ^a^	8.1 ^A^	8.1 ^b^	15.7 ^C^	9.7 ^c^	9.7 ^B^
*Listeria monocytogenes* 67	2.0 ^a^	2.0 ^A^	4.8 ^c^	9.3 ^C^	4.0 ^b^	4.8 ^B^
*L. monocytogenes* 74	1.7 ^a^	2.0 ^A^	4.8 ^c^	9.3 ^C^	4.0 ^b^	4.8 ^B^
*Listeria inocua* LI0001	2.0 ^a^	4.0 ^A^	4.0 ^b^	4.8 ^B^	4.0 ^b^	4.8 ^B^
*Bacillus cereus* 1	4.0 ^a^	4.1 ^A^	8.1 ^b^	8.7 ^B^	8.1 ^b^	8.1 ^B^
*B. cereus* 9	4.7 ^a^	4.7 ^A^	9.7 ^b^	9.3 ^C^	4.8 ^a^	8.1 ^B^
*Clostridium perfringens* Clpe0001	5.8 ^a^	7.8 ^A^	9.7 ^b^	9.7 ^B^	9.7 ^b^	9.7 ^B^
*Escherichia coli* 31	8.0 ^a^	18.7 ^A^	8.7 ^b^	18.7 ^A^	9.8 ^c^	19.7 ^A^
*E. coli* 22	8.1 ^a^	18.7 ^B^	9.4 ^b^	15.7 ^A^	9.7 ^b^	19.7 ^B^
*E. coli* 26	8.0 ^a^	18.7 ^A^	9.8 ^b^	19.7 ^A^	9.7 ^b^	19.7 ^A^
*E. coli* 34	8.0 ^a^	18.7 ^A^	13.7 ^b^	19.3 ^A^	8.4 ^a^	19.3 ^A^
*Klebsiella pneumonie* 003	8.4 ^a^	18.7 ^A^	9.4 ^b^	19.3 ^A^	9.7 ^b^	19.7 ^A^
*Salmonella typhimurium* 63 s	8.0 ^a^	16.2 ^A^	9.7 ^b^	19.7 ^B^	9.7 ^b^	19.7 ^B^
*S. typhimurium* 235	8.0 ^a^	16.8 ^A^	9.4 ^b^	19.7 ^B^	9.7 ^b^	19.7 ^B^
*Salmonella enteritidis* 61 s	6.7 ^a^	18.7 ^A^	9.7 ^b^	19.7 ^A^	9.7 ^b^	19.7 ^A^
*Pseudomonas aeruginosa* PA0001	8.0 ^a^	18.7 ^A^	9.7 ^b^	19.7 ^A^	9.7 ^b^	19.7 ^A^
*Pseudomonas fluorescens* ATCC13625	8.4 ^a^	18.7 ^A^	8.7 ^a^	19.7 ^A^	9.7 ^b^	19.3 ^A^

Statistical analysis was performed by one-way ANOVA using the Tukey’s *post hoc* test: different letters in the same row indicate statistical significance (*p* ≤ 0.05)

The high antimicrobial efficacy of Japanese quince, sea buckthorn and cranberry juices after neutralisation may arise from their content of flavonoids and anthocyanins. This is supported by the available literature data. It has been proven that polyphenolic compounds contained in cranberry may act as anti-adhesive agents in preventing and inhibiting the adherence of pathogens, which could have a beneficial effect, *e.g*., in treatment of urinary tract infections [30]. Other studies reported that polyphenolic rich extracts from Japanese quince and sea buckthorn showed high antimicrobial activity [7, 28].

## Conclusions

The analysis of antioxidant and antibacterial properties of various fruit juices led to several interesting findings. The highest antioxidant properties were determined for wild rose juice, followed by juices from chokeberry and Japanese quince. However, the highest antimicrobial activity against all tested strains of both Gram-positive and Gram-negative bacteria was identified in juices from cranberry, Japanese quince and sea buckthorn. These juices were characterised by the rich content of polyphenols (TP and FBBB assays), except cranberry juice, which had a significant content of anthocyanins. The high antimicrobial activity of Japanese quince, sea buckthorn and cranberry juices was the result of polyphenols and their low acidity, which – as demonstrated in earlier studies – arises from the presence of vitamin C and phenolic acids. Wild rose, chokeberry and elderberry juices, despite their high antioxidant properties, showed antimicrobial activity only against Gram-positive strains (except *Enterococcus faecalis* and *C. perfringens*). Wild rose and chokeberry juices had the highest total flavonoid content, while elderberry juice had the highest anthocyanin content. The lowest antioxidant properties were determined for noni juice, which showed an antimicrobial effect only against some Gram-positive bacterial strains. This fact may be a consequence of the presence of other bioactive compounds, *e.g.*, iridoids. Further studies, including the structure of polyphenols and other bioactive compounds demonstrating antimicrobial activity, should be conducted. Considering all the findings, we conclude that bioactive compounds in Japanese quince, sea buckthorn and cranberry juices could be used as potential sources of natural antioxidants and antimicrobials. Fruit extracts as new functional products (novel foods) can be an effective tool promoting health, considering the bacterial resistance to numerous antibiotics. The bioactive compounds of the analysed juices can be used not only for the prevention of non-communicable chronic diseases but also as natural preservatives in the food processing industry. The important issue is also the quality of fruit and juices, which may depend on storage, processing and packaging condition [35].

## Supplementary Information

ESM 1 (DOCX 25.9 kb)

## Data Availability

Not applicable.

## Declarations

### Human and Animal Participants

This article does not contain any studies with human or animal subjects.
