# Supplementary material for: Antioxidant and Antimicrobial Properties of Selected Fruit Juices
Source: Plant Foods Hum Nutr. 2022 Jul 13;77(3):427–35. doi: 10.1007/s11130-022-00983-2 (PMC9463271; doi:10.1007/s11130-022-00983-2)
Supplement: Supplementary file 1 — (DOCX 25.9 kb) [file 11130_2022_983_MOESM1_ESM.docx]

SUPPLEMENTARY MATERIAL

**Article title:** Antioxidant and antimicrobial properties of selected ~~berry juices and vitamin C-rich~~ fruit juices.

**Journal name**: Plant Foods for Human Nutrition

**Author names:** Dariusz Nowak^*^, Michał Gośliński, Lucyna Kłębukowska

Affiliation and e-mail address of the corresponding author:

^*^ Department of Nutrition and Dietetics, Faculty of Health Sciences, Ludwik Rydygier Collegium Medicum in Bydgoszcz, Nicolaus Copernicus University in Toruń, Dębowa 3, 85-626 Bydgoszcz, Poland; e-mail: d.nowak@cm.umk.pl

**Materials and methods**

**Materials**

Seven commercial NFC (not from concentrate) juices were selected for analyses. Three juices were made from fruit berries grown in Poland: elderberry (*Sambucus nigra* L.), chokeberry (*Aronia melanocarpa* (Michx.) *Elliott*) and cranberry (*Vaccinium macrocarpon* Aiton). Three juices, rich in vitamin C, were made from fruit grown in Poland: sea buckthorn (*Hippophae rhamno*ides L., cultivated in north-eastern Poland), wild rose (*Rosa canina* L., central Poland) and Japanese quince (*Chaenomeles japonica* (Thunb.) Lindl. ex Spach., eastern Poland). The seventh juice was produced from exotic fruit of noni (*Morinda citrifolia* L. from French Polynesia). All the juices were cold pressed from whole fresh fruits and submitted to mild continuous ﬂow pasteurisation at a temperature not exceeding 85°C. Before analysis the samples were refrigerate stored, no more than one week. The juices were naturally turbid, without any additives, which means that water was not added either.

**Methods**

The pH of the tested samples was measured with a glass electrode (Hanna Instruments, Olsztyn, Poland) at room temperature.

**Soluble Solids Content**

The soluble solids content of samples was measured with a laboratory refractometer RL-3 (PZO, Warsaw, Poland), at 20^◦^C, in a way similar to the method used by Rubio-Arraez et al. [1].

**Antioxidant Capacity**

**DPPH Assay**

The antioxidant capacity of the fruit juices was determined by a modified Yen and Chen method, using 0.1 mmol/L methanol solution of 1,1-diphenyl-2-picrylhydrazyl (DPPH, Sigma-Aldrich, St. Louis, MO, USA) [2]. This method is widely used to test the antioxidant capacity of fruit, vegetables and juices. Advantages of the DPPH assay were previously described [3–5]. The procedure was as follows: an amount of 0.1 mL of a sample was added to 2.9 mL of DPPH solution and mixed. The absorbance was measured on a Rayleigh UV-1800 V/VIS spectrophotometer at 517 nm after 30 min of incubation at room temperature in the dark. For each juice, samples were analysed in three replicates and the results were used to calculate an average value. The percentage of DPPH scavenging was calculated using Equation (1):

% scavenging = [(A_DPPH_ – A_juice_)/A_DPPH_] × 100 (1)

where A_DPPH_ is the absorbance of the DPPH blank solution and A_juice_ is the absorbance

of the sample solution.

The resultant value was then substituted into an equation of a previously prepared 6-hydroxy-2,5,7,8-tetramethylchromane-2-carboxylic acid (Trolox-Sigma-Aldrich) calibration curve. The antioxidant capacity of the samples was expressed as milligrams of Trolox equivalents (Sigma-Aldrich) per litre of sample (mg Tx L^−1^).

***ABTS Assay*** The antioxidant capacity was determined by the Re et al. [6] method with small modiﬁcations. In the ABTS method, 2,2’-azinobis-(3-ethyl-benzothiazoline-6-sulfonic acid) diammonium salt (ABTS, Sigma-Aldrich, St. Louis, MO, USA) and potassium persulfate solutions were mixed and stored overnight at room temperature in the dark for 12–16 h. ABTS solution was diluted with methanol to an absorbance of 0.70 ± 0.02 at 734 nm.

After addition of 1.0 mL of diluted ABTS solution (A734 nm = 0.700 ± 0.020) to 0.01 mL of antioxidant compounds or Trolox standards in methanol, the absorbance was measured on a Rayleigh UV-1800 V/VIS spectrophotometer at 734 nm against methanol after 1 min. Quantiﬁcation was performed using a Trolox standard curve. The antioxidant capacity of the samples was expressed as milligrams of Trolox equivalents (Sigma-Aldrich) per litre of sample (mg Tx L^-1^).

**Phenolic compounds**

**Total Polyphenol Content**

The total polyphenol content (TP) of the samples was determined in the Folin–Ciocalteu assay (Sigma-Aldrich) [7]. First, 0.3 mL of a sample was placed in a 10-mL capacity tube, next 0.05 mL 2 mol/L Folin–Ciocalteu reagent (Sigma-Aldrich, St. Louis, MO, USA) and 0.5 mL 20% sodium carbonate solution were added. The mixture was diluted by addition of 4.15 mL distilled water and mixed. The absorbance was measured on a Rayleigh UV-1800 V/VIS spectrophotometer at 765 nm after 30 min incubation in the dark at room temperature. A calibration curve was performed with gallic acid. The results were expressed as milligrams of gallic acid equivalents per litre of sample (mg GAE L^-1^).

**Fast Blue BB Assay**

Fast Blue BB is a novel method described by Medina [8] to quantify the phenolic compounds through direct interaction of polyphenols with the Fast Blue BB (FBBB) reagent (4-benzoylamino-2,5-diethoxybenzenediazonium chloride hemi(zinc chloride) salt; Sigma-Aldrich) in an alkaline medium. This method demonstrates higher values of gallic acid equivalents (GAE) than the Folin–Ciocalteu assay does [8, 9]. An 0.2 mL aliquot of 0.1% Fast Blue BB reagent was added to 2 mL of samples and mixed for 1 min and 0.2 mL 5% sodium hydroxide was added. The absorbance was measured on a Rayleigh UV-1800 V/VIS spectrophotometer at 420 nm after 90 min of incubation in the dark at room temperature. The results are expressed as gallic acid equivalents per litre of sample (mg GAE L^−1^).

**Total Flavonoid Content**

The total flavonoid content was measured using the colorimetric assay developed by Kapci et al. [~~10,~~ 4]. Briefly, 0.3 mL of 5% sodium nitrite was added to 1 mL of sample at zero time. After 5 min, 0.3 mL of 10% aluminium chloride was added. At the 6th min, 2 mL of 1M sodium hydroxide was added. The mixture was diluted by addition of 2.4 mL distilled water and mixed. The absorbance was measured on a Rayleigh UV-1800 V/VIS spectrophotometer at 510 nm. The total flavonoids content was determined by a (+)-catechin (Sigma-Aldrich) standard curve and was expressed as milligrams of catechin equivalents per litre of sample (mg CAE L^-1^).

**Total Anthocyanins**

Total anthocyanins (TA) were determined by the pH diﬀerential method (AOAC Oﬃcial Method 2005.02) [~~24~~ 10]. Juices were diluted according to appropriate dilution ratios (1 part sample and 4 parts buﬀer) by adding both 0.025 mol L^-1^ KCl (pH 1.0) or 0.4 mol L^-1^ CH_3_COONa·3H_2_O (pH 4.5) buﬀer solutions (Avantor Performance Materials). Samples were mixed and left in the dark for 30 min. Absorbance was measured on a Rayleigh UV-1800 V/VIS spectrophotometer at 520 nm and 700 nm, and the results were calculated using the following formula (2):

A = [(A_520_ − A_700_)_pH1.0_ − (A_520_ − A_700_)_pH4.5_] (2)

where A_520_ is the absorbance measured at 520 nm and A_700_ is the absorbance measured at 700 nm, at pH 1.0 and 4.5, respectively.

Total anthocyanins were expressed as milligrams of cyanidin-3-mono-glucoside equivalents per litre of sample (mg CGE L^-1^ juice [~~24~~ 10].

Molar extinction coeﬃcient = 26.900 L mol^-1^ mL^-1^ and molecular weight = 449.2 g mol^-1^.

**Antimicrobial activity of juices**

The antimicrobial activity in all the analysed juices was determined with the agar well diffusion method according to previous studies [~~9, 25~~ 11, 12]. Diameters of the inhibition zones for the growth of test Gram-positive and Gram-negative strains were identified. The test strains originated from a collection of strains maintained at the Department of Industrial and Food Microbiology of the University of Warmia and Mazury in Olsztyn. Surface cultures (105 CFU mL^-1^) of the test strains were started on sterile Petri plates filled with 20 mL Mueller–Hinton agar medium (Merck). Next, wells of 10 mm size diameter were made with sterile cork borer into agar plates containing the bacterial inoculum and filled with the analysed juices, each in an amount of 0.7 mL. The plates were incubated at optimal temperature (30°C or 37°C) for 24 h. In the case of the *Clostridium perfringens* strain, incubations were carried in anaerobic conditions. After the incubation, the diameters of the inhibition zones for the growth of the test strains around the wells were determined. The experiment was replicated thrice.

All the analysed juices were characterized by low pH (from 2.5 cranberry juice to 3.8 noni and elderberry juices). Therefore, in order to eliminate the inhibitory effect of this environmental factor on the tested strains, juices were neutralised with a sterile NaOH solution to pH 6.7-7.2 to study their antimicrobial activities in parallel.

**Minimal inhibitory concentration and minimal bactericidal concentration**

For juices that showed antimicrobial activity against the tested Gram-negative and Gram-positive strains (sea buckthorn, quince and cranberry), the minimal inhibitory concentration (MIC) and the minimal bactericidal concentration (MBC) in relation to the test strains were determined. MIC was determined by the dilution method on the Mueller-Hinton Broth medium (Merck), while the MBC was determined by the plate method on the Mueller-Hinton Agar medium (Merck) [~~26, 27~~ 13, 14].

**Test strains used in the study**

The following strains were submitted to tests:

Gram-positive: *Staphylococcus aureus* G3, *Staphylococcus aureus* 2G, *Staphylococcus aureus* 01, *Enterococcus faecalis* 24, *Enterococcus faecalis* 11, *Enterococcus faecalis* 07, *Listeria monocytogenes* 67, *Listeria monocytogenes* 74, *Listeria inocua* LI0001, *Bacillus cereus* 1, *Bacillus cereus* 9, *Clostridium perfringens* Clpe0001.

Gram-negative: *Escherichia coli* 31, *Escherichia coli* 22, *Escherichia coli* 26, *Escherichia coli* 34, *Klebsiella pneumonie* 003, *Salmonella* *typhimurium* 63s, *Salmonella* *typhimurium* 235, *Salmonella* *enteritidis* 61s, *Pseudomonas aeruginosa* PA0001, *Pseudomonas* *fluorescens* ATCC13625.

**Statistical analysis**

The results were statistically analysed by calculating the mean and standard deviation. The interpretation of the results was performed with MS Excel Analysis ToolPak software (Microsoft, Redmond, WS, USA) applying one-way analysis of variance (ANOVA) and the Tukey’s post hoc test: different letters in the same row or column in the tables indicate statistical significance (at least p ≤ 0.05).

**References**

1. Rubio-Arraez S, Capella JV, Castello ML, Ortola MD (2016) Physicochemical characteristics of citrus jelly with non cariogenic and functional sweeteners. J Food Sci Technol 53:3642–3650. https://doi.org/10.1007/s13197-016-2319-4
2. Yen G, Chen HY (1995) Antioxidant activity of various tea extract in relation to their antimutagenicity. J Agric Food Chem 43:27–32. <https://doi.org/10.1021/jf00049a007>
3. Apak R, Gorinstein S, Bohm V, Schaich K, Ozyurek M, Guclu K (2013) Methods of measurement and evaluation of natural antioxidant capacity/activity (IUPAC Technical Report). Pure Appl Chem 85:957–998. <https://doi.org/10.1351/PAC-REP-12-07-15>
4. Kapci B, Neradova E, Cizkova H, Voldrich M, Rajchl A, Capanoglu E (2013) Investigating the antioxidant capacity of chokeberry (Aronia melanocarpa) products. J Food Nutr Res 52:219–229
5. Nowak D, Gośliński M, Wojtowicz E (2016) Comparative analysis of the antioxidant capacity of selected fruit juices and nectars: chokeberry juice as a rich source of polyphenols. Int J Food Prop 19:1317–1324. <https://doi.org/10.1080/10942912.2015.1063068>
6. Re R, Pellegrini N, Proteggente A, Pannala A, Yang M, Rice-Evans C (199) Antioxidant activity applying an improved ABTS radical cation decolorization assay. Free Radic Biol Med 26:1231–1237. <https://doi.org/10.1016/S0891-5849(98)00315-3>
7. Singleton VL, Orthofer R, Lamuela-Raventos RM, Analysis of total phenols and other oxidation substrates and antioxidants by means of Folin–Ciocalteu reagent. Methods Enzymol 299:152–178. <https://doi.org/10.1016/S0076-6879(99)99017-1>
8. Medina MB (2011) Determination of the total phenolics in juices and superfruits by a novel chemical method. J Funct Foods 3:79–87. <https://doi.org/10.1016/j.jff.2011.02.007>
9. Nowak D, Gośliński M, Przygoński K, Wojtowicz E (2018) Antioxidant properties and phenolic compounds of vitamin C-rich juices. J Food Sci 83:2237–2246. <https://doi.org/10.1111/1750-3841.14284>
10. Lee J, Durst RW, Wrolstad RE (2005) Determination of total monomeric anthocyanin pigment content of fruit juices, beverages, natural colorants, and wines by the pH diﬀerential method: Collaborative study. J AOAC Int 88:1269–1278. <https://doi.org/10.1093/jaoac/88.5.1269>
11. Oikeh EI, Omoregie ES, Oviasogie FE, Oriakhi K (2016) Phytochemical, antimicrobial, and antioxidant activities of different citrus juice concentrates. Food Sci Nutr 4:103–109. https://doi.org/[10.1002/fsn3.268](https://dx.doi.org/10.1002%2Ffsn3.268)
12. Raza H, Shehzad MA, Baloach A, Ikram RM (2019) Antioxidant and antimicrobial activity of fruit juices. Int J Agric Sustain 1:94–103. https://doi.org/[10.33411/IJASD/2019010307](http://dx.doi.org/10.33411/IJASD/2019010307)
13. Balouiri M, Sadiki M, Ibnsouda SK (2016) Methods for in vitro evaluating antimicrobial activity: A review. J Pharm Anal 6:71–79. <https://doi.org/10.1016/j.jpha.2015.11.005>
14. Synowiec A, Gniewosz M, Bączek K, Węglarz Z (2011) Antimicrobial properties of water-ethanol extract from the leaves of bilberry (*Vaccinium myrtillus* L.) [in Polish]. Bromat Chem Toksykol 3:656–661
